# The "Goldilocks Effect" in Cystic Fibrosis: identification of a lung phenotype in the *cftr* knockout and heterozygous mouse

**DOI:** 10.1186/1471-2156-5-21

**Published:** 2004-07-27

**Authors:** J Craig Cohen, Lennart KA Lundblad, Jason HT Bates, Michael Levitzky, Janet E Larson

**Affiliations:** 1 Departments of Medicine and Physiology, Louisiana State University, School of Medicine, New Orleans, LA, 70112 USA; 2 The University of Vermont, Vermont Lung Center, Burlington, VT 05405-0075, USA; 3 Ochsner Children's Research Institute, Ochsner Clinic Foundation, New Orleans, LA 70121, USA; 4 Department of Clinical Physiology, Malmö University Hospital, Lund University, Malmö S-205 02 Sweden

## Abstract

**Background:**

Cystic Fibrosis is a pleiotropic disease in humans with primary morbidity and mortality associated with a lung disease phenotype. However, knockout in the mouse of *cftr*, the gene whose mutant alleles are responsible for cystic fibrosis, has previously failed to produce a readily, quantifiable lung phenotype.

**Results:**

Using measurements of pulmonary mechanics, a definitive lung phenotype was demonstrated in the *cftr*-/- mouse. Lungs showed decreased compliance and increased airway resistance in young animals as compared to *cftr*+/+ littermates. These changes were noted in animals less than 60 days old, prior to any long term inflammatory effects that might occur, and are consistent with structural differences in the *cftr*-/- lungs. Surprisingly, the *cftr*+/- animals exhibited a lung phenotype distinct from either the homozygous normal or knockout genotypes. The heterozygous mice showed increased lung compliance and decreased airway resistance when compared to either homozygous phenotype, suggesting a heterozygous advantage that might explain the high frequency of this mutation in certain populations.

**Conclusions:**

In the mouse the gene dosage of *cftr* results in distinct differences in pulmonary mechanics of the adult. Distinct phenotypes were demonstrated in each genotype, *cftr*-/-, *cftr +/-,* and *cftr*+/+. These results are consistent with a developmental role for CFTR in the lung.

## Background

Cystic fibrosis (CF) is a progressive disease primarily affecting the intestines, lungs, and pancreas. The gene responsible for CF was identified in 1989 [1] as coding for the cystic fibrosis transmembrane conductance regulator (*cftr*), a membrane chloride channel. CF is one of the most common autosomal recessive diseases in Caucasians with a carrier rate of 3–4% [2], and is characterized by recurrent infection and chronic inflammation. Recently it was found that infants with CF demonstrate changes in forced expiratory volume in 1 second (FEV_1_), functional residual capacity (FRC), and other parameters of lung function prior to the onset of recurrent infection [3-5].

Soon after the CF gene was discovered, a knockout mouse was developed. This mouse demonstrates subtle changes in epithelial cell phenotype, including alterations in secretory glycoconjugates and changes in secretory vesicles [6]. Monocytic infiltrates and altered lung mechanics have also been found [7]. Unfortunately, the *cftr* knockout mouse does not develop overt lung disease, which has severely limited its usefulness. However, the availability of new methods for pulmonary testing in rodents [8,9] now presents the opportunity to re-examine the *cftr* knockout mouse for functional lung changes. In the present study, therefore, we examined pulmonary function in young adult *cftr* -/-, *cftr* +/-, and *cftr*+/+ S489x mice in an effort to establish a lung phenotype.

## Results

### Effect of *cftr* gene dosage on pressure-volume (PV) curves

**Figure 1:**
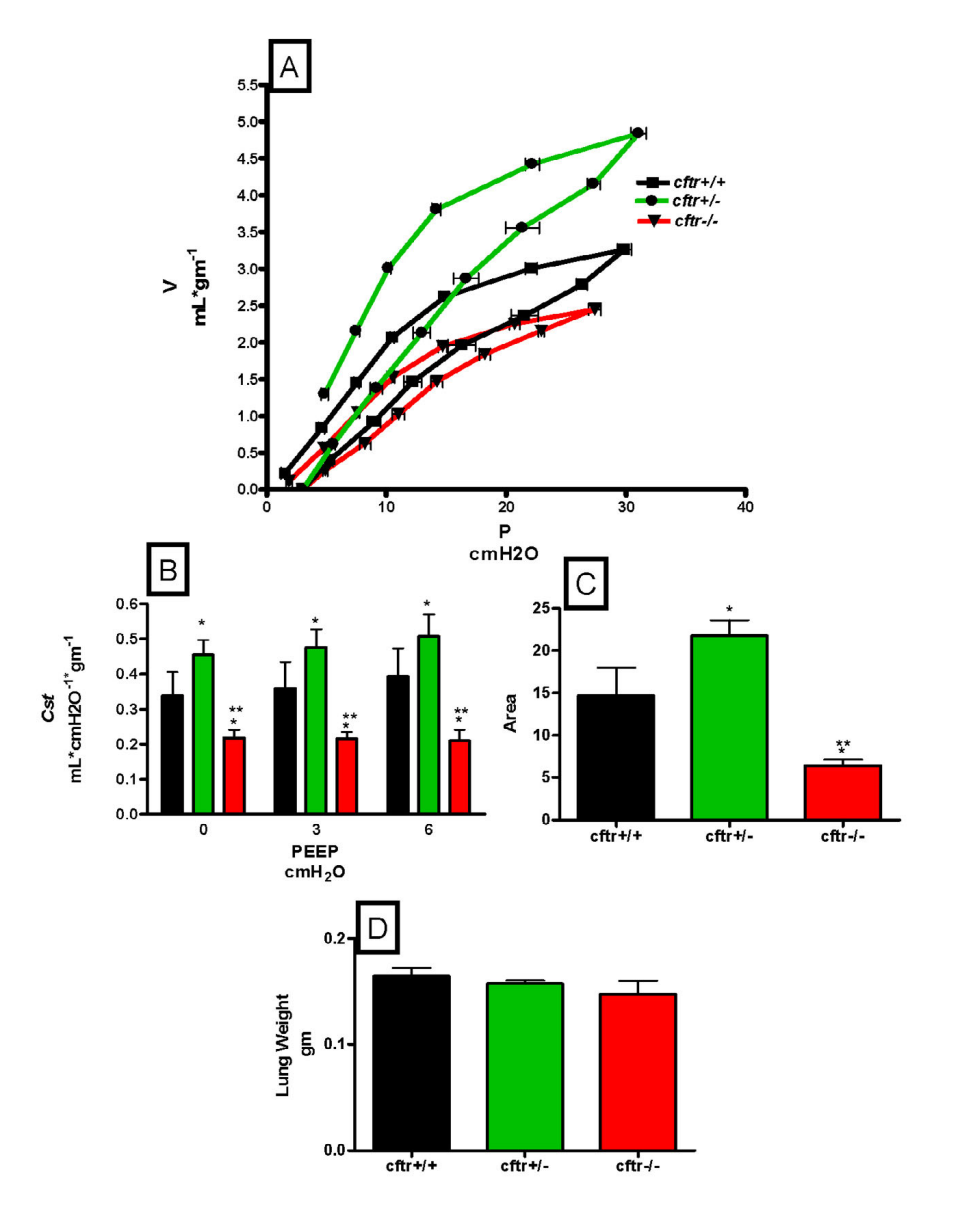
**Relationship between *cftr* genotypes and *PV* curves**. Littermates from 30–60 days of age were genotyped and individuals with *cftr*+/+ (Black), *cftr*+/- (Green), and *cftr*-/-(Red) were evaluated using pressure volume curve analysis at Peeps of 0, 3, and 6. A: *PV* curve at PEEP 3; B: Calculated *Cst* for all *PV* curves; C: Calculated hysteresis for PV curves in A. All measures were corrected individually for lung weight. Error bars are +/- standard deviation. *p < 0.05 when compared to *cftr*+/+ and **p < 0.05 when compared to cftr+/-.

Routine evaluation of dynamic lung function employs the stepwise variation in air volume on both the inflation and deflation phases of a single breath. Measurement of airway pressures at each step results in the classic pressure-volume (*PV*) curve which is dependent upon both lung structure and interfering pathology. *PV* curves were measured in triplicate, starting from positive end-expiratory pressure (PEEP) values of 0, 3, and 6 cmH_2_O in S489X mice at 30–60 days of age following genotyping for the normal and mutant *cftr* alleles. The 3 cmH_2_O PEEP curves obtained for each genotype are presented in Figure 1A. Note that these *PV* curves all begin at *V* = 0 ml, which is the FRC defined by the 3 cmH_2_O PEEP. The *PV* curves obtained at PEEP levels of 0 and 6 cmH_2_O were similar.

The static compliance (*Cst*) of the lungs, which reflects elastic recoil at a given pressure, was calculated from the slopes of the *PV* curves. As shown in Figure 1B, the static compliance of the homozygous knockout lung was significantly decreased compared to the homozygous normal lung (p < 0.01)). Furthermore, *Cst* was significantly reduced in both *cftr*+/+ (p < 0.001) and *cftr*-/- (p < 0.001) as compared to age-matched *cftr*+/- mice. Hysteresis was altered among the 3 genotypes (Figure 1C). A statistically significant increase in hysteresis was observed in both *cftr*+/- (P < 0.0001) and *cftr*+/+ mice (p < 0.05) compared to *cftr*-/- mice. These data suggest the presence of a gene dosage effect in which an altered lung structure in the heterozygous animals leads to an elevated compliance relative to the two homozygous animals. Lung weights were measured and no statistically significant differences were observed among the three genotypes (Figure 1D).

### Airway mechanics of *cftr* deficient lungs

**Figure 2:**
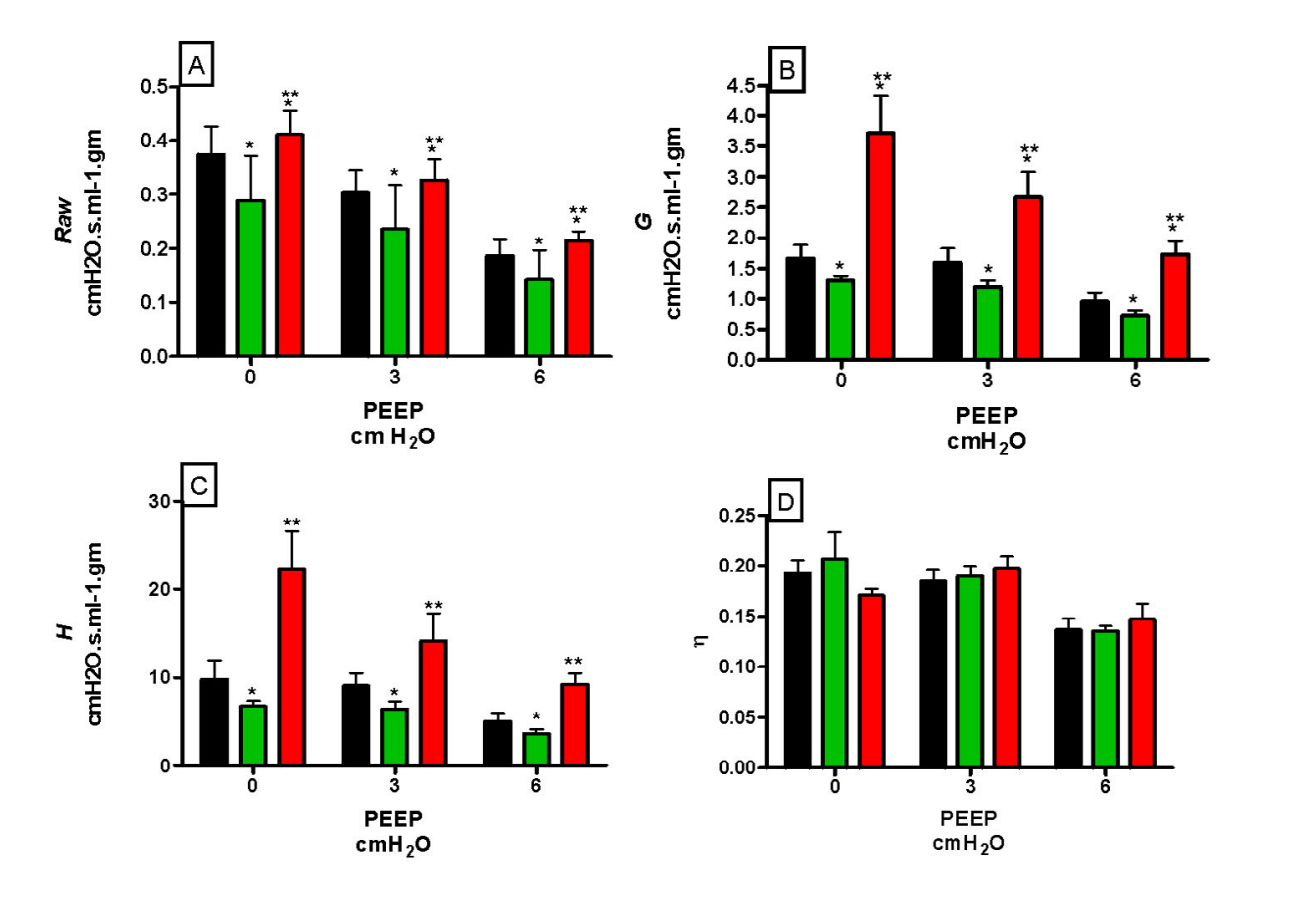
**Variation in respiratory mechanic among *cftr* genotypes.** Values for *Raw*(A), *G*(B), *H*(C), and *η*(D) were determined by fitting the constant-phase model to measurements of *Zrs* from *cftr*+/+ (Black), *cftr*+/- (Green) and cftr-/- (red) genotypes. All measures were normalized by multiplication by lung weight. Error bars are +/- standard deviation. *p < 0.05 when compared to *cftr*+/+ and **p < 0.05 when compared to cftr+/-.

We applied the forced oscillation method to the mice and determined respiratory mechanical input impedance [10,11]. We fit the constant-phase model of respiratory mechanics [12] to impedance (*Zrs*) and determined values for airway resistance (*Raw*), tissue damping (*G*), and tissue elastance (*H*). Figure 2 shows that *Raw*, *G*, and *H* were significantly reduced in the *cftr*+/- mice as compared to both *cftr*+/+ and *cftr*-/- animals (Panels A, C, & D) at all PEEP levels. *Raw* also decreased with PEEP in a similar fashion in all three genotypes. In contrast, *Raw*, *H* and *G* were significantly increased in the *cftr*-/- mice compared with *cftr*+/+ and *cftr*+/-, and showed a greater dependence on PEEP (Panels A, C & D). The ratio *G*/*H*, termed hysteresivity (both the low and high frequency), was not significantly affected by either genotype or PEEP (Panel E). Thus, the absence of either 1 or 2 copies of the *cftr* gene had significantly different effects on the phenotype of the lung. Paradoxically, the absence of only one *cftr* copy resulted in a greater lung compliance (lower elastance) than if neither or both copies were present.

## Discussion

The usefulness of the *cftr* knockout mouse as a model of cystic fibrosis has been severely limited by its failure to demonstrate readily measurable lung disease, the primary cause of morbidity and mortality in humans [13]. However, in the present study use of sophisticated measurements of lung function revealed a functional lung phenotype in the knockout mouse (Table 1); the complete absence of *cftr* in the lung of young adult animals resulted in decreased *Cst* and *η* and increased *Raw*, *G* and *H* as compared to normal littermate controls.

**Table 1:** Relative effect of genotype on lung function compared to that observed in the *cftr*+/+ mouse.

**Genotype**	***PV* curve position**	***Cst***	***PV* Hysteresis**	***Raw***	***G***	***H***	***η***
***cftr*+/-**	←	↑	↑	↓	↓	↓	↔
*cftr*-/-	→	↓	↓	↑	↑	↑	↔

A particularly intriguing further observation was that *Cst* and hysteresis in *cftr*+/- mice was significantly higher than in *cftr*+/+ animals while *G* and *H* were decreased. As this was not associated with any pathology such as emphysema, we conclude that it represents a functionally different lung from that of the *cftr*+/+. Our data thus reveal a remarkable inverse correlation between the effect of one and two non-functional copies of the *cftr* gene.

What do these data mean in terms of lung structure? The knockout animal has cystic fibrosis by definition, and our data now show it to also have lung disease manifest as a reduced compliance and increased resistance. These changes could reflect changes in the intrinsic mechanical properties of the parenchyma, or simply a reduction in lung volume. The former effect could include alterations in the biophysical properties of the air-liquid interface in the lungs, and would be expected to result in a change in *η*[14]. Indeed, because *cftr* is a chloride channel and is thought to be involved in water balance, a change in surface tension in the lung, and consequently in *η*, might be expected. However, as shown in Figure 2 and Table 1, although *G* and *H* both increase, they do so in the same proportion so there is no significant change in *η* between the three *cftr* genotypes. On the other hand, lung weights were not different among the different groups of mice, so the decreased compliance and increased resistance of the *cftr*-/- animals was not simply due to their having smaller lungs than control animals. This suggests that the parenchymal structure in the lungs of the homozygous and heterozygous animals were organized differently, in a manner that affected *G* and *H* similarly.

As documented in numerous publications, the mouse strain used in the present study does not develop chronic inflammatory disease up to the age (30–60 days) used in this study (for review see [15]). On the other hand, Broaches-Carter et al. [16] have shown that *cftr* levels are highest in the developing lung and decrease 75-fold at birth. *In utero* over-expression of *cftr* has also been shown to affect lung growth and development[17], and the severity of disease in the knockout mouse has been shown to be influenced by genetic background [18]. These data thus suggest that *cftr* may affect the early development of the lung in a manner that is affected by the interaction of other genes.

Are there any functional consequences for increased lung compliance in the heterozygous *cftr* animals? Interestingly, there is no decrement in lung function in human heterozygotes [19-21]. Also, the heterozygote frequency for CF in humans is higher than expected, likely reflecting a selective advantage because there is no evidence for genetic drift [22,23]. Indeed, selective advantage in CF has been proposed to reflect resistance to tuberculosis, influenza and cholera [24]. When one looks in nature for other examples of heterozygous advantage, the sickle cell trait which confers resistance to malaria [25] is perhaps the only such recognized genetic trait in humans. In Norway rats, a single Mendelian gene controls resistance to Warfarin, an anticoagulant used to control rat populations; homozygous wild-type rats are killed by Warfarin and homozygous mutant allele animals are highly susceptible to vitamin K deficiency [26]. The results of the present study indicate that a similar selective advantage may pertain to *cftr*, something we term a "Goldilocks Effect". That is, while two defective copies of the gene are detrimental and two normal copies are satisfactory, one normal and one defective gene may results in an optimal dosage for lung development.

Further studies of pulmonary mechanics in *cftr* knockout mice should reveal additional genetic loci that modulate the influence of *cftr* on lung growth and development. Corresponding studies in humans should be useful in evaluating the effect of therapies on reversing altered pulmonary function in the CF patient.

## Conclusions

Using sophisticated techniques to a evaluate rodent pulmonary function; a distinct, readily quantifiable lung phenotype was identified in the *cftr* knockout mouse. In addition, the *cftr*+/- mouse had a distinguishable pulmonary function phenotype from that observed in either the homozygous normal or mutant genotype mice. These data are consistent with CFTR-dependent, physiologic changes in the structure and function of the lung.

## Methods

### Mouse strain

The S489X mouse 5^th^ generation backcross to C57Bl/6 has been maintained by random mating for the past 8 years. This colony has a 100% mortality rate among *cftr* knockouts by 45 days of age unless the animals are placed on an elemental liquid diet and corncob bedding upon weaning [27]. Mice 30–60 days of age from our S489X mouse colony were genotyped for the normal and mutant *cftr* alleles. Age and litter matched *cftr*+/+ and *cftr*+/- were used for each *cftr*-/- mouse examined. Six animals were included in each group. All experiments were approved by the animal care and use committee.

### Pulmonary function tests

The mice were anesthetized with intra-peritoneal pentobarbital (90 mg/kg) and the trachea was dissected free of surrounding tissue and cannulated with a 20-gauge cannula. The animals were then connected to a small animal ventilator (*flexiVent*, SCIREQ Inc. Montreal, PQ, Canada) and ventilated with a tidal volume of 10 ml/kg; inspiratory:expiratory ratio of 66.67%, respiratory rate of 150 breaths/minute, and maximum pressure of 30 cmH_2_0. PEEP was controlled by submerging the expiratory limb from the ventilator into a water trap. Each animal was paralyzed with pancuronium bromide (0.5 mg/kg) and allowed to equilibrate on the ventilator until spontaneous breathing ceased (5 minutes).

#### Respiratory mechanics

To measure *Zrs*, mechanical ventilation was interrupted and the animal was allowed to expire against the set level of PEEP for 1 s. We then applied an 8 s broad-band volume perturbation signal to the lungs with the *flexiVent*, after which ventilated was resumed. This was repeated at PEEP levels of 0, 3 and 6 cmH_2_O. The volume perturbation signal consisted of the superposition of 18 sine waves having frequency spaced roughly evenly over the range 0.25 Hz to 19.625 Hz. *Zrs* was calculated from the displacement of the ventilator's piston and the pressure in its cylinder as described previously [10,11]. Correction for gas compressibility as well as resistive and accelerative losses in the *flexiVent*, connecting tubing and the tracheal cannula were performed as described previously [28] using dynamic calibration data obtained by applying volume perturbations through the tubing and tracheal cannula first when it was completely closed and then when it was open to the atmosphere.

We interpreted the measurement of *Zrs* in terms of the constant phase model [12]



where *Raw* is a frequency independent Newtonian resistance reflecting that of the conducting airways[29], *Iaw* is airway gas inertance, *G* characterizes tissue damping, *H* characterizes tissue stiffness (elastance), *i* is the imaginary unit, *α* links *G* and *H*, and *f* is frequency. We also calculated a quantity known as hysteresivity (*η* = *G/H)*, which is believed to increase when regional heterogeneities develop in the lung [30]. *Raw*, *G* and *H* were all normalized by multiplication by lung weight.

#### PV curves

Starting at the *FRC* defined by the PEEP, the *flexiVent* was programmed to deliver seven inspiratory volume steps for a total volume of 0.8 ml followed by seven expiratory steps, pausing at each step for 1 s. Plateau pressure (*P*) at each step was recorded and related to the total volume (*V*) delivered to produce a quasi-static *PV* curve. *Cst* was calculated from the slope of each curve [31], and was normalized by division by lung weight.

*Zrs* measurements at each PEEP level and *PV* curves were obtained in triplicate. Data were statistically evaluated using paired t-test with p < 0.05 being taken as significant
